# A model of collaboration for the implementation of problem-based learning in nursing education in South Africa

**DOI:** 10.4102/curationis.v40i1.1765

**Published:** 2017-08-28

**Authors:** Mahlasela A. Rakhudu, Mashudu Davhana-Maselesele, Ushonatefe Useh

**Affiliations:** 1 School of Nursing Sciences, North-West University, South Africa; 2 Office of the Rector, North-West University, South Africa; 3 School of Post Graduate Studies and Research, North-West University, South Africa

## Abstract

**Background:**

The idea of collaboration between key stakeholders in nursing education for the implementation of problem-based learning (PBL) may have far-reaching implications for the institutions and students.

**Main objective:**

To develop a model of collaboration to facilitate the implementation of PBL in nursing education.

**Methodology:**

An exploratory sequential design was used. Qualitative data were collected from purposively recruited nurse educators from three universities in South Africa offering PBL and nurse managers from all the three hospitals in North West Province where PBL students are placed for clinical learning. A questionnaire was used to obtain data from respondents who were conveniently recruited. Model development, concept analysis, construction of relationships, description and evaluation were followed.

**Results:**

This model has six elements: higher education and nursing education (context), institutions initiating PBL, clinical services, colleges affiliated to PBL universities, students and healthcare users (recipients), champions in PBL (agents), effective implementation of PBL (terminus), collaboration (process) and commitment, communication, trust and respect (dynamics).

**Conclusion:**

Collaboration in implementing PBL can be a functional reality in the delivery of quality educational experiences and has far-reaching implications for the institutions and students. The implementation of the model in South African nursing education institutions may be necessary in the light of the revision of the preregistration qualifications.

**Recommendations:**

Managerial commitment, training of collaborators on PBL and collaboration skills, memorandum of agreement, monitoring and evaluation are critical. More research is required to pilot the model and evaluate collaboration in implementing PBL at different levels of operations.

## Introduction

Student-centred collaborations can be a functional reality in the delivery of quality higher educational experiences (Brown 2009). The idea of collaboration between centres of excellence, academic institutions and clinical practice in the implementation of problem-based learning (PBL) in nursing education may have far-reaching implications for the institutions, departments and students.

## Collaboration in nursing education

Collaboration is a substantive idea repeatedly discussed in healthcare circles. Though its benefits are well validated, collaboration is seldom practised (Zamanzadeh et al. 2014).

Hall-Lord, Theander and Athlin (2013) highlighted collaboration between universities and clinical placements as a weak point of nursing education. After the transfer of nursing education to universities, the students’ time for learning in the clinical setting has decreased in many Western countries, as has the nurse lecturers’ supervision of students in direct patient care (Hall-Lord et al. 2013). This resulted in the lecturers’ role changing from supervising students in ‘hands-on nursing’ to focusing more on nursing theory and research.

Although in South Africa 4000 hours are prescribed by the regulatory body, reality is uncertainty over the clinical role of nurse lecturers persists. Collaboration is a crucial component of all aspects of the clinical school development, as it is through this on going and continuous process that a common vision and common goals and realities are developed and maintained. This warrants the development of collaboration approaches in education of the future healthcare workforce.

Miller et al. (2015) remind us that strong collaborative partnerships between academia and practice need to move beyond short-term clinical learning of students into meaningful long-term relationships that enhance both the education and practice environments (Morton 2013). The healthcare organisations seeking recognition for excellence in service delivery acknowledge the value of strong relationships with nursing programmes (Morton 2013). Clinical nursing education is core to the nursing profession and, therefore, collaboration of academia and clinical services must be upheld in implementing PBL. For this reason, development of a model of collaboration in implementing PBL was necessary based on the opinions of nurse educators and nurse managers.

The needed collaboration is influenced by both changes occurring in the healthcare system and limited availability of resources, namely financial and human.

Collaboration with key stakeholders, as well as centres of excellence in PBL and healthcare service providers, especially where students are placed for clinical learning, will enhance the mentoring and empowerment of the students and nurse educators as well as nurse managers and other preceptors in PBL. For this reason, the development of a collaboration model for the implementation of PBL based on the nurse educators’, nurse managers’ and preceptors’ opinions was necessary. In a collaborative model, partners share knowledge, expertise and resources. Thus, nurse educators need not remain in isolation while learning to use PBL. Collaborative partnerships are imperative, particularly in the view of pending budget cuts for schools of nursing (Bentley & Seaback 2011).

According to Bedwell et al. (2012), managers in various work settings rely on collaborative partnerships to achieve institutional goals. These collaborative processes take many forms such as intra-professional and interdisciplinary coalitions, strategic alliance and joint ventures (Bedwell et al. 2012). Therefore, the school of nursing sciences, in an effort to facilitate the implementation of PBL in nursing education, developed a model of collaboration based on the empirical data from nurse educators and managers and concept analysis.

## Problem-based learning

Higher education institutions are mandated to improve undergraduate education by implementing initiatives to target instructional methods, re-examine curricula and apply innovative technologies to better engage students with content. PBL is regarded as the most innovative teaching learning modality conceived and implemented in medical and health sciences (Ertmer 2013; Hung & Loyens 2012). Its effectiveness in enhancing application of knowledge, problem solving, collaborative and self-directed learning skills and high-order thinking has been documented (Khatiban & Sangestani 2014; Mansor et al. 2015; Yang & Yang 2013).

PBL provides an environment for promoting these skills (Borhan 2012; Shin & Kim 2013; Yew & Goh 2016). Its active and student-centred instructional strategies can help the students utilise their knowledge and skills in new situations (Niemer, Pfendt & Gers 2010; Williams and Beattie 2008). In a typical PBL setting, learning is triggered by a problem that needs resolution. The teacher acts as a facilitator to guide student learning through the learning cycle.

PBL has proved to be successful in enhancing students’ active participation and problem-solving and critical thinking skills (Borhan 2012; Chan 2012; Shin & Kim 2013). According to Williams and Beattie (2008) and Sockalingam and Schmidt (2011), in PBL students are encouraged to identify own knowledge and skills and apply them in real situations by combining previous knowledge to achieve specific goals. Therefore, PBL is perceived as an approach to close the theory–practice gap in nursing education (Applin et al. 2011; Rakhudu 2011; Sangestani & Khatiban 2013).

Given these benefits of PBL, the school of nursing sciences in North-West University (NWU), a historically under-resourced institution, implemented PBL in 2002. On exploration of nursing students’ experiences and perceptions in 2008, the students voiced the need to strengthen PBL implementation by collaboration between the university and the clinical practice settings. This was reiterated during external programme evaluation in 2008 wherein collaborative partnerships between academia and clinical practice for effective implementation of PBL were recommended (NWU 2008). This prompted the need to develop a model of collaboration in implementing PBL in nursing education.

## Main objective of the study

The main objective was to develop a collaboration model for effective implementation of PBL in nursing education. The following specific objectives were applicable to achieve the above:

- To explore and describe the opinions of the nurse educators, nurse managers and preceptors regarding collaboration in implementing PBL in nursing education.
- To measure and describe the opinions of nurse educators, nurse managers and preceptors regarding collaboration in implementing PBL.
- To explore the emerging concepts derived from data analysis.
- To develop a collaborative model for effective implementation of PBL in nursing education.

## Model development methodology

The model development consisted of two phases, namely empirical (explorative) and model development. Phase 1 used a sequential exploratory mixed-method design to obtain the opinions of nurse educators and managers on collaboration in implementing PBL in nursing education. Phase 2 used a four-phased approach described by Chinn and Kramer, namely concept analysis, construction of relationships, description and evaluation of the model (Chinn & Kramer 2011).

## Empirical phase: Fieldwork

### Methodology

In the explorative phase, a sequential, exploratory, mixed-method design (Creswell & Plano-Clark 2011) was employed with the aim of exploring and describing the opinions of nurse managers and educators on collaboration in implementing PBL in nursing education. Semi-structured individual interviews (*n* = 11) and focus group interviews (*n* = 33) were used to collect data from purposively sampled nurse educators from three (*n* = 3) out of five (*n* = 5) universities in South Africa offering PBL in nursing education and all the three (*n* = 3) hospitals in North West Province where PBL students are placed for clinical learning. This was followed by a descriptive survey using a self-administered questionnaire from 96 respondents who were conveniently recruited to measure the opinions on collaboration in the implementation of PBL from the participants from the selected institutions. Given the fact that qualitative design was conducted first, a sequential method had to be employed.

### Sampling

The following criteria were used to recruit participants who would best answer the questions (Creswell 2014; Munhall 2012):

- Nurse educators: The educator must be registered as a nurse educator with South African Nursing Council (SANC) and teaching at a South African university offering PBL and possess at least two years’ experience at a higher education learning institution.
- Nurse managers: Managers should be employed in the provincial hospital in the North West Province where PBL students are placed for clinical learning and should have two years’ experience in a managerial position.
- Universities**:** The university should be offering PBL to the undergraduate nursing programme.

### Data collection

Individual semi-structured interviews and focus group discussions were conducted from June 2011 to May 2012, while the descriptive surveys were conducted from June 2012 to July 2012. Realisation of the qualitative sample was achieved after interviewing 11 participants and six focus group discussions (*n* = 33). From the quantitative component, 120 questionnaires were personally delivered and also sent through emails to the participants. Overall, 96 were returned, representing 80% return rate.

### Data analysis

In this study, the statements from collected data were grouped and given codes for easy identification later in the study (Creswell 2014). These codes acted as guidelines for this study to help the researcher conduct data analysis simultaneously with data collection.

### Ethical consideration

The study was approved by the university ethics committee from North-West University (Ethics number: NWU-00033-11-A9). Permission from North West Provincial Department of Health and management of participating hospitals and universities was obtained. Volunteers who fitted the criteria were recruited to participate and requested to give written consent after expectations of their participation were explained. The purpose of the study was explained clearly to the institutional contacts and individuals within the study. Participation was voluntary. The identities of the institutions and individuals were kept confidential by use of codes.

### The results

The results indicated the importance of intra-professional, inter-professional and inter-institutional collaboration. The results were utilised to form the basis for the development of the concept ‘collaboration’ and theory development by means of concept definition, classification and generation of relationship statements in order to design a model for effective implementation of PBL.

### Quality measures

In qualitative research, the researcher works with truths that are socially situated. Thus, measures for ensuring trustworthiness of the findings by Guba (in Krefting 1991) were utilised. Table 1 depicts the strategies that were used to ensure truth value.

**TABLE 1:** The strategies used to ensure truth value.

Criterion	Description
Member checking	The findings was taken back to participants and ensuring that they agree (Munhall 2012).
Reference adequacy	The transcribed interviews, the research protocol and re-order’s protocol were in the final report, in order to increase reference adequacy.
Authority of the researcher	The researcher has experience of qualitative research in Master’s studies and was guided by a highly rated researcher who is acting as a promoter.

*Source*: Authors’ own work

In the quantitative component, content validity was checked with collaboration experts from Johns Hopkins University faculty members and McMaster University and NWU as well as Medical Research Council statisticians. The tool was piloted with 10 participants from the participating universities and hospitals.

## Model development phase

The study aimed at the development of a model of collaboration for implementing PBL in nursing education. The method of theory generation as explained by Chinn and Kramer (2011) was adopted and utilised in this study. The sequence of four phases described by Chinn and Kramer was followed, namely concept analysis, construction of relationships, model description and evaluation of the model. The model development process is described in the following section.

## Concept analysis

A search of various dictionaries, subject textbooks and research publications was performed. A literature search between the years 2000 and 2015 in the databases of the Cumulative Index to Nursing and Allied Health Literature, Medline and PsycINFO was conducted. The inclusion criteria of the search were as follows: publication should be in English and published from 2000 to 2015. At first, 490 full-text articles and 360 abstracts were retrieved. However, many of the retrieved documents were the same articles indexed in multiple databases. After removing those repetitive documents, 275 articles remained in the study database. Of the retrieved articles, the title and abstract of only those articles that had defined or analysed for the concept of collaboration were selected. Finally, 36 articles from nursing and allied health were included in the final analysis of the concept of collaboration. The articles were reviewed for trends that would reflect the current knowledge for collaboration as a concept. The search key terms were ‘collaboration’, ‘problem based learning’ and ‘nursing education’. The purpose of this concept analysis was to better understand and define collaboration as it relates to the implementation of PBL.

The following adapted activities of the framework of Rodgers and Knalf (2000) were utilised to guide the analysis of the concept of collaboration:

- Definition of the concept of interest generally or dictionary definition.
- Identification and selection of appropriate realm (setting and sample) for data collection.
- Data collection relevant to identify the attributes of the concepts along with surrogate terms, references and antecedents.
- Data analysis regarding the above characteristics of the concept.
- Identification of model case of the concept.

This framework was used to clarify the critical attributes of collaboration, identifying elements needed to be present (antecedents) for the concept to occur, distinguishing the concept from the multitude of related terms and consequences and to assist in the development of a comprehensive understanding to facilitate the application of the concept in nursing education practice (Petri 2010).

## Concept classification

After concept analysis, concept classification followed using the survey list developed by Dickoff, James and Wiendenbach (1968). The survey list assisted to clarify prescription or activities necessary to teach the desired behaviours of the situation producing theory (Dickoff et al. 1968). Table 2 displays the components of the survey according to Dickoff et al. (1968).

**TABLE 2:** Components of survey list according to Dickoff et al. (1968).

Component	Description
Agents	Who performs the activity?
Recipient	Who is the recipient of the activity?
Context	In what context is the activity performed?
Procedure	What is guiding procedure of the activity?
Dynamics	What is the energy source of the activity?
Terminus	What is the end point of the activity?

*Source*: Authors’ own work

## Construction of relationship statements

A relationship statement links two or more concepts together. Polit and Beck (2010) refer to a relationship as a bond or association between two or more variables. The two types of relationship statements used include associative and causal statements. Burns and Grove (2009) describe associative relationships as relations that identify concepts that occur or exist together in the real world; thus, when one concept changes, the other concept changes too. These relationships are part of theory and can be tested through research. The authors explain the causal statements or statements that demonstrate a cause–effect relationship.

In this research study, after concepts had been identified, relationship statements were developed asking questions such as:

- Are the concepts stated alone or independent?
- Are the concepts occurring together or associative?
- Are the concepts related to each other?
- Are the concepts influencing each other?

The answers to those questions will indicate the types of relationships emerging to impact structure to the theory and facilitate understanding (Chinn & Kramer 2011).

The identified essential and related attributes were used to construct relationships described in the next section.

The relationship statements formulated for this model of collaboration in implementing PBL are given below:

- Collaboration in implementing PBL is influenced by the context wherein PBL in education is occurring, namely higher education, nursing education and clinical healthcare context. These boundaries represented these contexts.
- Successful collaboration in implementing PBL is dependent on dynamics such as commitment (managerial, organisational and individual), communication (formal and informal), cooperation, respect and trust both at the organisational and individual levels.
- Collaboration is an interactive, dynamic and beneficial process occurring in phases.
- This dynamic process of collaboration leads to effective PBL implementation.
- Students and healthcare users or consumers are at the core of this collaboration in implementing PBL as they are the ultimate recipients of PBL in nursing education. Having identified the benefits of PBL, the students and healthcare service consumers are also the ultimate beneficiaries of collaboration in implementing PBL in nursing education.
- Collaboration has a lot of spin-off benefits for the participating institutions and individuals.

## Description of the model

Once the concepts have been defined and classified and the relationship statements constructed, the description of a model to represent the theoretical concepts can be done. The model for collaboration in implementation of PBL has six main elements: higher education, nursing education and health care services (context), institutions initiating PBL, clinical services, colleges affiliated to PBL universities, students and healthcare users (recipients), centres of excellence in PBL (agents), effective implementation of PBL (terminus) collaboration, (process) and commitment, communication, cooperation, trust and respect (dynamics).

**FIGURE 1:**
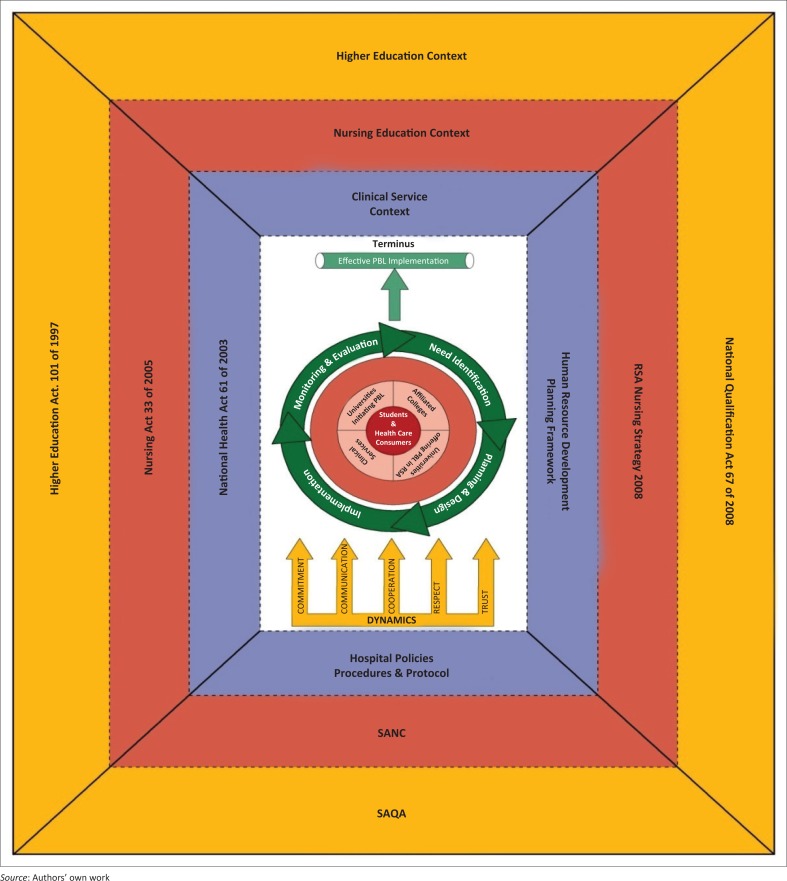
A model of collaboration in implementation of problem-based learning in nursing education.

Figure 1 depicts a model of collaboration in implementation of PBL in nursing education.

The symbolic meanings of the schematic presentation are as follows:

- The frames around the model represent the context in which collaboration will be taking place, namely higher education, nursing education and clinical context.
- The figure in the centre represents all that occurs in collaboration when implementing PBL.
- The inner and middle circles represent the recipients of the activity (collaboration) while the outer circle represents the agents. The outer circular arrows represent the procedure or the process of collaboration, which are cyclical and imply that if the objectives of collaboration are not achieved, the process should be repeated, starting with analysis.
- The arrow from the process to the terminus indicates the direction of the process to the goal or end point.
- The pointed arrows at the bottom indicate the dynamics that maintain or sustain the activity, namely commitment, communication, collegiality, trust and respect. These are critical forces that influence the process, agents and recipients, and goal or outcome of the process.

The model description is based on the method of Chinn and Kramer (2011) and consists of the following components: (1) overview of the model, (2) the purpose of the model, (3) the structure consisting of assumptions on which the model is based, (4) concepts definition and (5) relationship statements

A schematic representation in Figure 1 depicts a model of collaboration for effective implementation of PBL. The model is based on the premise that collaboration is necessary and beneficial to all stakeholders in implementing PBL in nursing education (made apparent from the research). The data collected from the sequential exploratory mixed-method study indicate that nurse educators in PBL universities, nurse managers and preceptors from clinical settings where PBL students are placed indicate the need and benefits for collaboration in implementing PBL in nursing education. In addition to this is the fact that there is a great pressure for nursing education institutions (NEIs) by authorities to produce nurse graduates who are competent to: (1) function effectively within a multidisciplinary team, organisation and community, (2) solve problems appropriately using critical and lateral thinking, (3) communicate effectively, (4) collect, analyse and critically evaluate information and (5) explore educational and career opportunities and be lifelong learners.

PBL education institutions provide an environment for promoting achievement of these skills. To ensure that these skills do not remain visionary benchmarks, attempts should be made by educational institutions to operationalise them by embedding the outcomes in curricular activities. Thus, a model of collaboration for effective implementation of PBL is deemed necessary.

The model proposes the collaborative activities that occur through four sequential stages, namely need analysis, planning and design, monitoring and evaluation. These four processes feed on each other and overlap. During the course of the processes, the collaborators will learn how to function more adequately on behavioural, cognitive and affective levels of collaboration.

## Purpose of the model

This proposed model will be used as a frame of reference to facilitate the design of collaboration intervention to benefit the participants to effectively implement PBL in nursing education.

## The structure of the model

The structure of the model gives the overall form of the conceptual relationships within it (Chinn & Kramer 2011). The structural form of a model aids in understanding the central relationships between concepts, their order of occurrence and how they interact. This model was based on the following elements: assumptions, concept definition, relationship statements and the nature of the structure.

## Assumptions of the model

Assumptions are the accepted truths on which the model is based (Chinn & Kramer 2011). They are not only closely related to relationship statements but also reflect the values underlying the model. For this reason, it is important to make them explicit, so that they can be understood on their own terms and from the perspective of the model that the researcher intended. It will also enable critique of the model by those who hold different views. The assumptions underlying the main concept (Collaboration) and other concepts used in the model are explained hereunder:

- Collaborators in PBL implementation bring different skills, knowledge and talents.
- Participants in collaboration are driven by the same goal, vision and mission, and this needs to be carefully crafted to align the activities.
- Organisational climate and culture of the PBL institutions can influence the collaboration activities.
- Collaboration in implementing PBL is dynamic and brings up changes in the collaborative partners and their clientele, namely students and healthcare consumers.

## Concepts definition

The identification and definition of the key related concepts are here given to clarify the structure of the model. The concepts were identified through the process of concept analysis and opinions of the participants and were classified and clarified through six elements of Dickoff et al. (1968). These concepts include:

- Higher education, nursing education and clinical context (context or framework);
- Centres of excellence in PBL (agent);
- Universities initiating PBL, other universities offering PBL in the region, affiliated colleges, clinical services, students and healthcare users or consumers (recipients);
- Collaboration process that is dynamic, transformational and beneficial consisting of need analysis, joint planning and design, execution or implementation and monitoring and evaluation (procedure);
- The outcome of collaboration, which was effecting implementation of PBL (terminus) and;
- Dynamics or underlying powers of collaboration including commitment, communication, cooperation, respect and trust (dynamics).

Context: The context is viewed as the framework and multipurpose environment in which collaboration in implementing PBL will take place. This enables the activity to be viewed in relation with other things. As the model will be operationalised within the context of higher education, nursing education and clinical settings, the realities of those environments must be taken into consideration by participants when collaboration is planned or designed.

The following section describes the context within which collaboration will take place, namely higher education, nursing education and clinical contexts.

The higher education: It is an environment that is dynamic and multidimensional in which nursing education is located and regulated. Norms and standards in this context influence nursing education. The context is regulated by the following legislative frameworks:

- *Higher Education Act, 1997 (Act 101 of 1997)*: which regulates higher education qualification and programmes by making provisions for quality assurance and promotion.
- *National Qualification Framework Act, 2008 (Act 67 of 2008)* that has been developed to establish the South African Qualification Authority (SAQA) and to provide for Quality Councils that constitute higher education and training quality assurers (South Africa 2008a).
- SAQA has set standards of the educational outcomes in the country and also designed Critical Cross Field Outcomes, which form a foundation for description of more specific outcomes in all the learning outcomes.

Nursing education: is the context wherein PBL education occurs. This context consists of a regulatory body of SANC and legal framework, which ensures the highest standards of nursing education and service delivery. Nursing Education Institutions (NEI’s) such as PBL universities and affiliated colleges are within this context. The universities affiliated with specific colleges are charged with the responsibility to maintain nursing education standards as stipulated by SANC. Therefore, in collaboration in implementing PBL, creation of an environment conducive to effective implementation is to occur within the boundaries of universities and the SANC guidelines and regulations. One critical act that influences this context is the *Nursing Act, 2005 (Act 33 of 2005)*.

This Act is a very important aspect of the nursing education context because it regulates the nursing profession and provides for matters related to nursing education and practice as well as SANC (South Africa 2005).

SANC is charged with accountability for setting standards of nursing care to the citizens of South Africa while the South African Nursing Strategy 2008 gives guidance on the operations and activities regarding nurse training within this context. The National Nursing Strategy is renewed on a five-year basis and focuses on producing competent degree and diploma graduates who are critical thinkers and effective decision makers.

Clinical healthcare: As a context, clinical healthcare context is a dynamic multipurpose environment that provides opportunities for PBL students to integrate theoretical components into practice. It provides PBL students with meaningful and authentic learning opportunities and experiences. In this context, multidisciplinary team members participate in education and training of PBL students in collaboration and this is to be created within the legal, moral, ethical and professional boundaries of healthcare services. The National Health Department obligates the Provincial and District healthcare services to participate in the training of nursing students as future healthcare human resources through the *National Health Act, 2003 (Act 67 of 2003)*. This context is regulated by various legislative frameworks as well as hospital policies and protocols.

Agents: Agents are collaborators who are expected to lead the collaboration through mentoring, development and capacity building in PBL activities. In this context, the agents are centres of excellence in PBL who have advanced knowledge, skills and expertise in PBL and collaboration.

Recipients: These are the following groupings that are to be monitored, developed and empowered on PBL, namely universities that are novices in PBL and affiliated colleges, other universities offering PBL, clinical services, students and healthcare users. Within the university where PBL education is initiated, and it is where intra-professional, inter-professional and inter-institutional collaboration occur.

Dynamics: Dynamics are the underlying powers and sources that initiate and maintain collaboration in implementing PBL and include commitment, communication, cooperation, respect and trust.

Process: It is a dynamic, cyclical, interactive and beneficial process consisting of need analysis, planning and design, implementation and monitoring and evaluation.

Effective implementation of PBL: This is the ultimate outcome or end point of collaboration to facilitate effective PBL implementation, which will benefit the nursing students and ultimately nursing.

## Evaluation of the model

The model was not evaluated by experts in this project as it will be done as a post-doctoral study. However, the critical reflection of this model was done according to Chinn and Kramer (2011) to help clarify how well it relates to theory, research or practice. These authors suggest ingredients for evaluation purposes as: clarity, simplicity, generality, accessibility and importance (Chinn & Kramer 2011).

The above five critical components that the researcher used to evaluate the model are discussed below.

Clarity: Semantic clarity and consistency were attained by using the same concept definitions in the same order throughout the model description. Clarity of the model was attained through concept analysis and empirical data from the participants’ opinions. Attributes and connotation of collaboration were identified through the Evolutionary Review of Rodgers and Knaff (2000) and through literature search.

Structural clarity was achieved through the survey list of Dickoff et al. (1968) and the six elements were used as the basis for describing the model.

Simplicity refers to the complexity of structural components and the relationships between categories (Chinn & Kramer 2011). The structure of the model is not too complex, in that it is easy to ascertain the relationships between the concepts and the intended outcomes of the model are also clearly indicated. The relationships between the concepts are clearly explained and straightforward.

Generality: The model was designed for the establishment of collaboration in implementing PBL for nursing education in NEIs. However, it can also be applied for the development of collaborative initiatives in nursing education and practice.

The model can be used in any educational situation where there is a need for collaboration with adaptation.

Accessibility: The model will be made available to NEIs initiating and offering PBL and clinical settings where data were collected through workshops and research conducted by the researcher.

The model will also be made accessible through publication in accredited journals and through presentation at seminars and workshops.

Importance: The importance of the model in nursing education relates to its practical value in the teaching arena. This model is deemed to be useful because it aims to address current shortages of skills, expertise and talents in PBL. The model will also promote effective utilisation of resources, namely sharing of decision-making processes, problem solving and accountability in nursing education or student issues.

## Limitations of the study

A limitation of this study is the restriction of the study to nursing education of a preregistration programme, which implies limited generalisations. The sample of nurse managers and preceptors was confined to the North West Province hospitals and clinics, where PBL students are placed for clinical learning as compared to nurse educators from three South African universities offering PBL. This was attributed to the fact that clinical services used by participating universities to place their PBL students were far apart and very costly to get the managers together in terms of finance and time. The empirical data from nurse managers were from one province, and therefore, the findings can only be transferable within the province. The model has not yet been tested.

## Recommendations

Recommendations for nursing practice: Clinical practice provides realistic and humane opportunity for PBL students to integrate theory into practice. Clinical service personnel should work jointly in education and training of the students from need identification to monitoring and evaluation of the PBL programme. It is important that the accredited providers of the practical component of PBL programme be educated and trained on PBL and collaboration as well as what it entails because this has a significant implication for collaborative practice.

Effective collaboration with healthcare providers requires that good relationships be maintained between academia and healthcare facilities and formal agreements should be in place detailing the roles and responsibilities of the collaborators.

Recommendations for nursing education: The model should be made available to the NWU Senate and SANC who regulate nursing education within the institution and nursing profession, respectively. The designed PBL programme, particularly its delivery mode, should be included in the university year book and SANC programme document. These recommendations are made in the light of the revision of the new professional qualifications in nursing education.

Recommendations for research: Higher education, nursing education and clinical healthcare settings are very dynamic and are characterised by changes influenced by political, economic, cultural and social factors in South Africa and globally. As the components of the model will not change, the detailed description of the model will require revisions to embrace the significant changes.

Much has been documented on collaboration, but a great deal is not yet clearly understood and requires more research, such as: (1) piloting the model and evaluating it, (2) collaboration in implementing PBL at different levels of operations, (3) cultural influences on collaboration, (4) criteria to assess effectiveness of collaboration and (5) development of an evaluation instrument of the model.

## Conclusion

This article dealt with the process of the development of collaboration for the implementation of PBL in nursing education in South Africa. The model development consisted of two phases, namely empirical (explorative) and model development. Phase 1 used a sequential exploratory mixed-method design to obtain the opinions of nurse educators and managers on collaboration in implementing PBL in nursing education. Phase 2 used the four-phased approach of Chinn and Kramer, namely concept analysis, construction of relationships, description and evaluation of the model. Description of the model was in accordance with Chinn and Kramer (2011) in terms of purpose and context, underlying assumptions, related concept definitions, relationship statements, and structure and process of collaboration in implementing PBL in nursing education.

The model suggests that collaboration with centres of excellence or champions of PBL is likely to promote mentoring and guidance on PBL curriculum development, implementation and evaluation. This type of collaboration is also likely to promote sharing of knowledge, skills and talents. Collaboration in implementing PBL has the potential of bridging the gap between nursing practice and academia and is a necessary prerequisite for assuring a qualified nursing workforce for the future and for positioning nurses to address emerging healthcare needs. Thus, this collaboration is vital for effective implementation of PBL, especially that nurse leaders in academia and practice settings have a long history of collaborating with one another for the purposes of enhancing nursing education, care and practice (Kirschling & Erickson 2010).
